# Polyphenol-Rich Propolis Extracts Strengthen Intestinal Barrier Function by Activating AMPK and ERK Signaling

**DOI:** 10.3390/nu8050272

**Published:** 2016-05-07

**Authors:** Kai Wang, Xiaolu Jin, Yifan Chen, Zehe Song, Xiasen Jiang, Fuliang Hu, Michael A. Conlon, David L. Topping

**Affiliations:** 1 College of Animal Sciences, Zhejiang University, Hangzhou 310058, China; kaiwang628@gmail.com (K.W.); chnfhjxl@163.com (X.J.); cgxllx@163.com (Y.C.); zehesong@gmail.com (Z.S.); xiasenjiang@163.com (X.J.); 2 CSIRO Food and Nutrition, Adelaide 5000, Australia; david.topping@csiro.au

**Keywords:** propolis, tight junctions, Caco-2, AMPK, ERK

## Abstract

Propolis has abundant polyphenolic constituents and is used widely as a health/functional food. Here, we investigated the effects of polyphenol-rich propolis extracts (PPE) on intestinal barrier function in human intestinal epithelial Caco-2 cells, as well as in rats. In Caco-2 cells, PPE increased transepithelial electrical resistance and decreased lucifer yellow flux. PPE-treated cells showed increased expression of the tight junction (TJ) loci occludin and zona occludens (ZO)-1. Confocal microscopy showed organized expressions in proteins related to TJ assembly, *i.e.*, occludin and ZO-1, in response to PPE. Furthermore, PPE led to the activation of AMPK, ERK1/2, p38, and Akt. Using selective inhibitors, we found that the positive effects of PPE on barrier function were abolished in cells in which AMPK and ERK1/2 signaling were inhibited. Moreover, rats fed a diet supplemented with PPE (0.3% in the diet) exhibited increased colonic epithelium ZO-1 expression. Overall, these data suggest that PPE strengthens intestinal barrier function by activating AMPK and ERK signaling and provide novel insights into the potential application of propolis for human gut health.

## 1. Introduction

Propolis is a plant-derived substance collected by honeybees (*Apis mellifera* L.) from various sources [1]. Abundant polyphenolic constituents, mainly flavonoids and phenolic acids, have been identified in propolis [2]. It has well-documented pharmacological activities, including antimicrobial, antioxidant, anti-inflammatory, immunomodulatory, and cardioprotective effects, and it is used widely as a health/functional food worldwide [3].

The intestinal mucosal barrier mainly consists of intestinal epithelial cells (IECs) on the luminal side and tight junctions (TJ) between IECs [4]. This barrier allows the transcellular transport of essential nutrients and controls the diffusion of luminal macromolecules and bacteria across the gut mucosa. TJ dysfunction or destruction is always accompanied by intestinal mucosal barrier disruption, leading to the penetration of xenobiotics/toxins into the lumen, which activates the intestinal immune system as can occur in inflammatory bowel disease (IBD) [5]. Despite several pharmacological therapies for IBD (which mainly rely on anti-inflammatory drugs), a fair number of patients still do not achieve full remission. Recent studies suggest that increasing intestinal barrier integrity has great therapeutic potential in IBD treatment [4,6].

TJs function to keep the intestinal physical barrier intact [7]. They are composed of multiple transmembrane proteins (e.g., occludin, claudins, and junctional adhesion molecule), and interact with cytosolic adaptor proteins, like zona occludens (ZO) proteins-1, 2, and 3. Cooperation between occludin and ZO-1 has a critical role in the maintenance of normal TJ structures and intestinal epithelial barrier function [8]. Recent evidence suggests that TJ permeability and TJ proteins are regulated by several intracellular signaling pathways, such as the AMP-activated protein kinase (AMPK) and mitogen-activated protein kinase (MAPK) cascades. The activation of these pathways depends on various physiological requirements and pathological challenges [9]. *In vitro* studies using the human intestinal epithelial cell line Caco-2 have highlighted their roles in the regulation of TJ permeability and expression of TJ proteins [10,11]. To evaluate the integrity of TJs, electron microscopy (including transmission electron microscopy (TEM) and scanning electron microscopy (SEM)) can be used to observe intercellular spaces and the ultrastructure between/within neighboring IECs [12,13]. Furthermore, TJ permeability through the paracellular pathway can be measured by transepithelial electrical resistance (TER) or tracer flux (e.g., using ruthenium red, FITC–dextran, or ^3^H-mannitol) [14,15].

There is growing interest in the development of novel therapeutic reagents against IBD that increase intestinal barrier function. Recent studies have shown that abundant dietary intake of polyphenol-rich foods, like green tea, coffee, berries, grapes, and other fruits/vegetables, has a beneficial effect on the maintenance of an intact intestinal barrier and increases TJ-related protein expression (occludin, claudin-1, and ZO-1) [6]. Polyphenol-rich propolis extracts (PPE) have been shown to have potent effects against various inflammatory diseases, including colitis [1,16]. The anti-bacterial properties of PPE have also been widely investigated and these studies suggest a role in the regulation of the gut microbiota [17]. However, it is not clear whether propolis has beneficial effects on the intestinal mucosal barrier.

In the present study, we studied the effects of PPE on intestinal barrier function in Caco-2 cell monolayers. We also tested AMPK and MAPK signaling in response to PPE treatment with respect to TJ integrity. In addition, rats were fed diets supplemented with PPE to investigate colonic TJ expressions.

## 2. Materials and Methods

### 2.1. Chemicals

Chemicals, including lucifer yellow (LY), alkaline phosphatase (AP)-conjugated secondary antibody (anti-rabbit IgG), *tert*-butyl hydroperoxide (tBHP), and 2,4,6-trinitrobenzenesulfonic acid (TNBS) were purchased from Sigma-Aldrich (St. Louis, MO, USA). Rabbit anti-ZO-1 and anti-occludin were purchased from Abcam (Cambridge, MA, USA). The selective inhibitors dorsomorphin (for AMPK signaling), PD98059 (for extracellular signal-regulated protein kinases, ERK, 1/2 signaling), SB203580 (for p38 signaling), and LY294002 (for Akt signaling) were obtained from Selleck Chemicals (Houston, TX, USA). All other reagents were purchased from Sangon Biotechnology (Shanghai, China), unless otherwise indicated.

### 2.2. Preparation and Extraction of Propolis Samples

A propolis sample (voucher specimen No. CP120820) was obtained from colonies of honeybees (*A. mellifera* L.) in the summer of 2012 from Zhejiang Province, China. The main plant origin was poplar (*Populus* spp.). The PPEs were obtained previously using ethanol as the solvent [18]. Raw propolis (100 g) was extracted by 95% (*v/v*) ethanol (1 L), sonicated at 40 °C for 3 h. The supernatant was collected and filtered to remove the residues. The raw propolis was extracted for three times. Then the supernatants were collected and evaporated in a rotary evaporator under a reduced pressure at 50 °C to evaporate the ethanol. Dried PPE were stored at −20 °C until further use. Their main chemical composition was determined by high-performance liquid chromatography (HPLC) and negative ion electrospray ionization mass spectrometry (ESI-MS). The total polyphenol content of PPE was 53.8 ± 0.9 g/chlorogenic acid equivalent/100 g. The leading polyphenolic constituents were chrysin, followed by galangin, kaempferol, and caffeic acid phenethyl ester [19].

### 2.3. Cell Culture

Human intestinal epithelial (Caco-2) cells were obtained from the Institute of Biochemistry and Cell Biology, SIBS, CAS (Shanghai, China) and maintained under standard cell culture conditions at 37 °C and 5% CO_2_ in a humidified incubator using high-glucose Dulbecco’s modified Eagle’s medium (Pierce Hyclone, Fremont, CA, USA), supplemented with 100 U/mL penicillin, 100 μg/mL streptomycin, and 10% (*v/v*) heat-inactivated fetal bovine serum (Gibco, Carlsbad, CA, USA). To measure intestinal barrier function, cells were placed on polycarbonate membranes in Transwell inserts (12-mm diameter, 0.4-μm pore size; Costar, Cambridge, MA, USA) for 14 days to induce cell growth and monolayer formation. The cells were refreshed and monitored regularly (every two days) by measuring TER. Cell viability was examined using a commercially available kit (cell counting kit-8; Dojindo, Kumamoto, Japan) [20,21].

### 2.4. Intestinal Barrier Function Determination

The intestinal barrier function was evaluated by measuring TER and unidirectional LY flux as previously described [21,22]. Various drugs were added to the apical side of the filter and the point of addition for each treatment was set to 0. TER values were read from a Millicell-ERS (Bedford, MA, USA). All readings were subtracted from the blank resistance for the well with culture medium only. For the paracellular permeability analysis, LY (100 μM) was added to the apical wells, and flux into the basal side, and assessed for 3 h LY incubation. The LY concentration on the basal side was determined using a fluorescence plate reader (BioTek, Winooski, VT, USA).

### 2.5. Animal Protocols

Eighteen male Sprague-Dawley rats (300–320 g) were obtained from the Laboratory Animal Services Centre, Zhejiang University. All rats were housed in cages in a room with a controlled temperature (22 ± 2 °C) and relative humidity (40%–60%) and a 12-h light–dark cycle throughout the study. The composition of rat chow was based on the AIN-93 diet (Table S1) [23]. The PPE was mixed with oil and added to the diet at 0.3% (*w/w*). The propolis dose was selected based on our previous rat studies and then calculated based on the rats daily food intake [19,24]. The rats were allowed to acclimate to their environment for seven days before experimentation. After the experiment began, rats were randomly assigned to one of three groups (*n* = 6 per group): a normal control group, TNBS colitis group, and PPE group. After two weeks, TNBS (50 mg/mL, dissolved in 50% ethanol solution) was injected at a dose of 100 mg/kg to the rats from the colitis group, following previous instructions [25,26]. The normal control and PPE groups received the same amount of ethanol solution (2 mL/kg), without TNBS during induction. All animals were killed 24 h later and three 1-cm segments of the distal colon were collected for specific analysis (gene expression, hematoxylin and eosin (HE) staining, and SEM). The animal study protocols were approved by the Animal Care and Use Committee of Zhejiang University, China (ethic approval code: AEC-150403).

### 2.6. Real-Time Quantitative Polymerase Chain Reaction (qPCR)

Total RNA was extracted with the RNA Pure Kit (Aidlab Biotechnologies Co., Ltd., Beijing, China) according to the manufacturer’s instructions. RNA samples were reverse-transcribed using a PrimeScript RT Reagent Kit (TaKaRa, Dalian, China). Quantitative PCR was performed using a 7500c Real-time PCR Detection System (Applied Biosystems, Carlsbad, CA, USA) with SYBR Premix Ex Taq (TaKaRa) following the manufacturer’s instructions. Expression of the housekeeping gene *Gapdh* was used for the normalization of expression levels. The Caco-2 primers are designed to flank introns with the Primer 5 software (Premier Biosoft, Palo Alto, CA, USA) by our group. Specificity of the primers was checked by the melting curve. The PCR products also have been tested by DNA sequencing and electrophoresed on the agarose gel. The primers sets were as follows: Occludin, 5′-GAGGTTTAGATTAGATTTCCGAC-3′ (F) and 5′-CACAACAAACTCCTTAGAACAAT-3′ (R); ZO-1, 5′-AGATGAACGGGCTACGC-3′ (F) and 5′-GGAGACTGCCATTGCTTG-3′ (R); *Gapdh*, 5′-AGGGATGATGTTCTGGAGAG-3′ (F) and 5′-TCAAGATCATCAGCAATGCC-3′ (R). Primer sets used in the rat study were obtained from a previous publication [27].

### 2.7. Immunoblot Analysis

Cellular proteins were lysed on ice in cell lysis buffer (150 mM NaCl, 0.5% NP-40, 10% glycerol, 2 mM DTT, 1 mM leupeptin, and 1 mM PMSF in 50 mM Tris-Cl, pH 7.5). Protein concentrations were determined using a BCA Protein Assay Kit (Beyotime, Nanjing, China). Equal amounts of cellular proteins (20 μg) were mixed with Laemmli sample buffer and boiled at 95 °C for 5 min, separated by SDS–PAGE, and transferred to PVDF membranes. Membranes were blotted using specific antibodies in combination with AP-conjugated anti-rabbit IgG antibodies. The immunoreactive protein bands on the membranes were developed using NBT/BCIP solution (18.75 mg/mL nitro blue tetrazolium chloride (NBT) and 9.4 mg/mL 5-bromo-4-chloro-3-indolyl phosphate toluidine salt (BCIP in 67% DMSO, *v/v*)) with 10 mL of color development buffer (100 mM Tris-Cl, pH 9.5, 50 mM NaCl, and 5 mM MgCl_2_). The Western blotting results were quantified using Quantity One software [20].

### 2.8. Immunofluorescence Staining

After treatment, Caco-2 cells cultured in laser confocal Petri dishes (Corning, Lowell, CA, USA) were washed in phosphate-buffered saline (PBS) three times, fixed with pre-cooled methanol and acetone (v:v = 1:1) solution for 30 min, and permeabilized with 0.5% PBS-Triton for 30 min. After incubation with 10% normal goat serum in PBS, immunofluorescence slides were incubated with occludin (1:500 dilution) and ZO-1 (1:200 dilution) overnight in a refrigerator. Then, the secondary FITC-conjugated goat anti-rabbit IgG (1:500 dilution, Mutisciences, Hangzhou, China) was applied for 1 h at 37 °C in the dark. After three rinses in PBS, the cell nuclei were stained with DAPI for 5 min. Finally, cells were visualized with a confocal laser microscope (Leica, TCS SP5, Wetzlar, Germany) and images were taken under an Olympus FLUOVIEW FV1000 Microscope (Tokyo, Japan).

### 2.9. Statistical Analysis

All data are presented as means and SD. The statistical analyses were performed in SPSS 16.0 (SPSS Inc., Chicago, IL, USA). The significance of the differences between all treatment groups was analyzed by one-way ANOVA followed by Duncan’s multiple range tests. Student's two-tailed *t*-tests were used for pairwise comparisons. *p* < 0.05 was accepted as statistically significant.

## 3. Results

### 3.1. Effects of PPE on TER and Lucifer Yellow Flux

**Figure 1:**
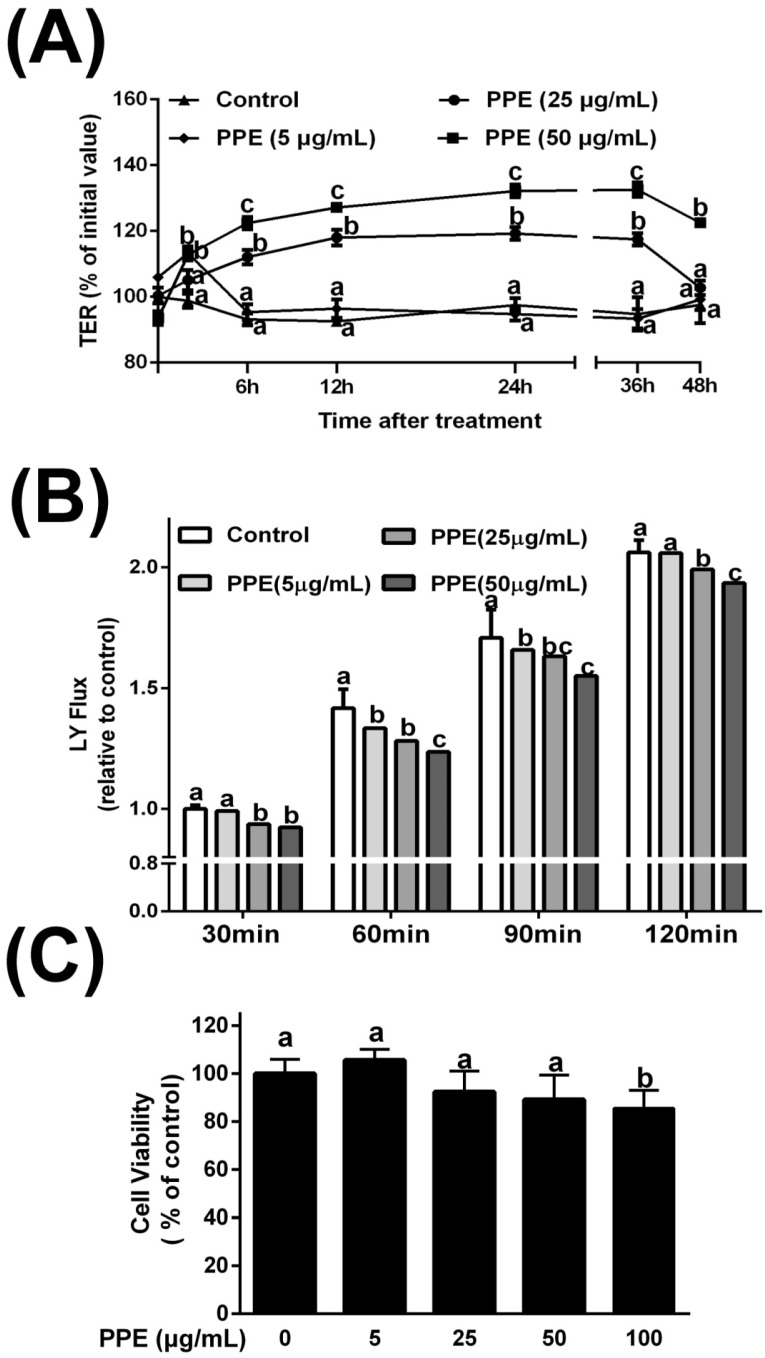
Effects of polyphenol-rich propolis extracts (PPE) on intestinal TJ permeability in Caco-2 cell monolayers. (**A**) Transepithelial electrical resistance (TER) in PPE-treated Caco-2 cell monolayers. Caco-2 cell monolayers were grown on 12-Costar Transwell filters for 14 days. TER was measured after treatment of Caco-2 monolayers with PPE (▲, 0 µg/mL, ◆, 5 µg/mL, ●, 25 µg/mL, ■, 50 µg/mL) at the indicated time points. The change in TER is expressed as the percentage change compared to the initial TER for each monolayer. The values represent the means ± SD (*n* = 3). Means sharing the same letter are not significantly different from each other (*p* < 0.05); (**B**) Unidirectional lucifer yellow (LY) flux in PPE-treated Caco-2 cell monolayers. LY flux was measured 120 min across Caco-2 cell monolayers after PPE exposure. Values represent means ± SD (*n* = 3) and are expressed as percentages of LY permeation. Means sharing the same letter are not significantly different from each other (*p* < 0.05); (**C**) Cellular viability was measured at 48 h post-PPE treatment using a cell counting kit-8 proliferation analysis. Results are expressed as % relative to control cells and data are given as means ± SD of three independent experiments, each performed in triplicate. Means sharing the same letter are not significantly different from each other (*p* < 0.05).

To evaluate the effects of PPE on intestinal epithelial TJ permeability, TER and LY flux were measured in Caco-2 cell monolayers. We found a progressive time- and dose-dependent increase, starting from 2 h after treatment with intermediate/high concentrations of PPE (25 and 50 μg/mL). TER values peaked at 36 h after 50 μg/mL PPE treatment, with a 32.2% gain with respect to the initial value. An intermediate concentration of PPE had a moderate effect on epithelial TJ permeability; 24 h treatment led to a 20.1% increase in TER (Figure 1A). Regarding paracellular permeability, the LY flux results showed similar trends to those observed for TER; PPE treatment (25 and 50 μg/mL) led to a dramatic decrease in LY flux values (Figure 1B). Only these two concentrations of PPE were safe for Caco-2 cells since they did not cause significant reductions in cell viability (Figure 1C). Since PPE treatment at a dosage of 50 μg/mL showed optimal effects, PPE (50 μg/mL) was administered at this dosage in subsequent *in vitro* experiments.

### 3.2. Effects of PPE Treatment on Gene Expression and Distribution of Tight Junction Proteins (*ZO-1* and *Occludin*) in Caco-2 Cell Monolayers

**Figure 2:**
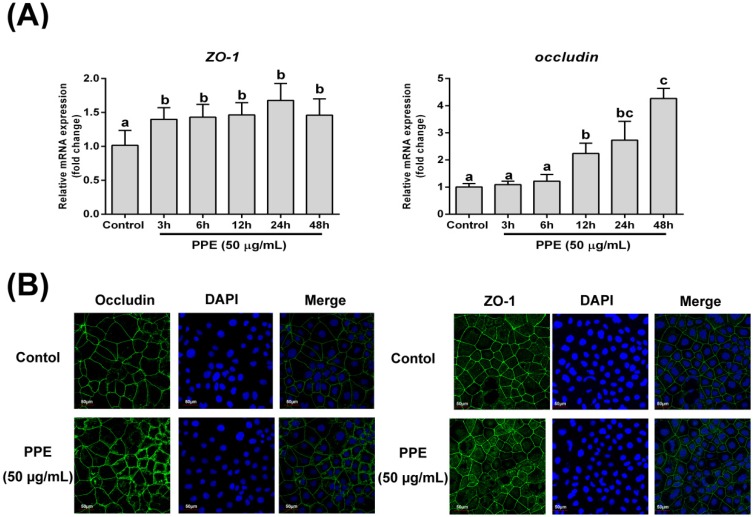
Effects of PPE treatment on gene expression, the distribution of tight junction proteins (ZO-1 and occludin) in Caco-2 cells. (**A**) Effect of PPE treatment on the mRNA expression of ZO-1 and occludin. Caco-2 cells were grown to confluence on 6-well plates and treated with PPE (50 μg/mL) for the indicated periods. ZO-1 and occludin mRNA expression levels were analyzed by qRT-PCR. Data are presented as means ± SD (*n* = 3). Means sharing the same letter are not significantly different from each other (*p* < 0.05); (**B**) Confocal microscopy images of immuno-stained tight junction proteins in confluent Caco-2 cells untreated or treated with PPE (50 µg/mL) for 48 h. Representative confocal microscopy images were obtained by fluorescent microscopy from three independent experiments after immunofluorescence staining of ZO-1 and occludin. 4′,6-Diamidino-2-phenylindole (DAPI) staining was performed to identify nuclei.

We next used real-time quantitative PCR to determine the expression of two important TJ genes, ZO-1 and occludin. As shown in Figure 2A, PPE (50 μg/mL) treatment led to increased expression of ZO-1 and occludin. The change in ZO-1 expression began at 3 h after PPE treatment, with a 48.9% increase at maximum at 24 h (*p* < 0.01). PPE showed a delayed, but stronger regulatory effect on occludin expression; significant induction was observed at 12 h after PPE treatment (*p* < 0.001) and a 4.7-fold change was observed at the end of the study period. We next used confocal microscopy to investigate the effects of PPE on TJ protein localization. As shown in Figure 2B, occludin and ZO-1 were appropriately localized to their respective intercellular junctions, in both control and PPE-treated cells. Next, we used TEM to observe alterations in TJ ultrastructure. A loss of TJ membrane fusion was observed in tBHP-treated cells with visible paracellular gaps, while PPE-treated cells exhibited extremely tight TJ structures (Figure S1).

### 3.3. PPE Treatment Activates AMPK and ERK Signaling in Caco-2 Cell Monolayers and Selective Inhibitors Block PPE-Induced TJ Regulation

**Figure 3:**
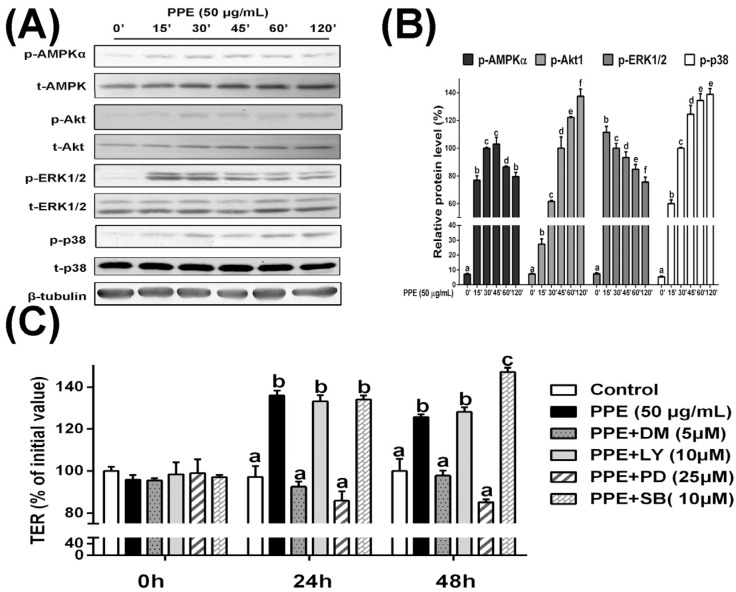
PPE treatment activates AMPK and ERK signaling in Caco-2 cell monolayers and selective inhibitors block PPE-induced TJ regulation. (**A**) Caco-2 cells were grown to confluence on six-well plates and treated with PPE (50 µg/mL) for the indicated periods. Whole cell lysates were collected and further subjected to an immunoblot analysis. Selective antibodies were used to detect the expression of phospho-AMPKα, total-AMPK, phospho-Akt, total-Akt, phospho-ERK1/2, total-ERK1/2, phospho-p38, and total-p38. β-Tubulin was used as a loading control. Representative Western blots are shown from three independent experiments; (**B**) The intensity of corresponding bands was measured by densitometry and normalized to β-tubulin. The values are the means ± SD (*n* = 3). Means sharing the same letter are not significantly different from each other (*p* < 0.05); (**C**) Caco-2 cell monolayers were grown on 12-Costar Transwell filters for 14 days. Cells were pretreated for 1 h with selective inhibitors: 5 µM DM (Dorsomorphin, for AMPK signaling), 10 µM LY (LY294002, for Akt signaling), 25 µM PD (PD98059, for ERK1/2 signaling), and 10 µM SB (SB203580, for p38 signaling) before treatment with PPE (50 µg/mL) for 24 h or 48 h. The change in TER is expressed as the percentage change compared to the initial TER for each monolayer. The values are the means ± SD (*n* = 3). Means sharing the same letter are not significantly different from each other (*p* < 0.05).

Next, to gain insight into the roles of roles of particular signaling pathways during PPE-mediated regulation of permeability, we determined the effects of PPE on the phosphorylation of AMPKα, Akt, ERK1/2, as well as p38. As shown in Figure 3A, Caco-2 cells incubated with PPE showed significantly increased phosphorylation levels of ERK1/2 (peaked at 15 min after PPE treatment), AMPKα (peaked at 45 min), Akt and p38 (both peaked at 120 min)(Figure 3A and Figure S2). These data encouraged us to examine the effects of signaling pathway inhibitors on TJ permeability. Dorsomorphin (a selective inhibitor of AMPK signaling), LY294002 (a selective inhibitor of Akt signaling), PD98059 (25 µM, a selective inhibitor of ERK1/2 signaling), and SB203580 (10 µM, a selective inhibitor of p38 signaling) were applied to Caco-2 cell monolayers before 50 µg/mL PPE treatment. As illustrated in Figure 3C, treatment of Caco-2 cell monolayers with either the AMPK signaling inhibitor dorsomorphin or the ERK1/2 signaling inhibitor PD98059, but not the Akt signaling inhibitor LY294002, counteracted the beneficial effects of PPE with respect to TER values. Surprisingly, we noticed that blocking p38 MAPK signaling via SB203580 led to further increases in TER, suggest that the role of the p38 MAPK pathway in PPE-mediated TJ permeability is different from other signaling pathways.

### 3.4. Effects of Oral Administration of PPE on Rat Colonic TJ mRNA Expression and Colon Morphological Changes

**Figure 4:**
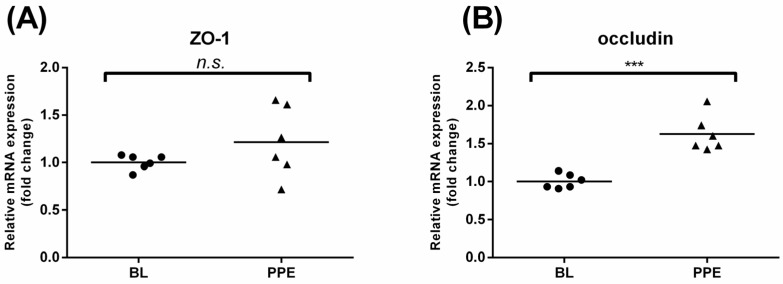
Effects of oral administration of PPE on rat colonic TJ mRNA expression. Distal colon tissues were collected from rats that were administered the control diet or control diet containing PPE (0.3% w/w). Effects of PPE administration on the mRNA expression of ZO-1 and occludin in rat distal colons. ZO-1 (**A**) and occludin (**B**) mRNA expression was analyzed by qRT-PCR. Data are presented as means ± SD (*n* = 6). *** *p* < 0.001, n.s., not significant.

We next asked whether PPE has similar beneficial effects on intestinal TJs *in vivo*. After 14 days of PPE administration, we found that PPE resulted in marked differences in the mRNA expression of occludin in the colon (*p* < 0.001 compared to control rats, Figure 4A). However, no significant change in ZO-1 expression was found (Figure 4B). Using HE staining and light microscopy, we also noticed that TNBS led to serious damage in the colon epithelium, with structural losses and the necrosis of the mucosa and submucosa, while PPE and control rats had quite similar, normal structures (Figure S3A). An ultrastructural analysis by scanning electron microscopy revealed that the microvilli of the colon epithelium were severely fractured by TNBS, unlike in the control and PPE groups (Figure S3B).

## 4. Discussion

Polyphenols, which contain more than 8000 identified compounds, are among the most numerous and widely distributed groups of substances in plants [28]. A growing number of studies have provided evidence for the health-promoting properties of polyphenols [29,30]. Dietary polyphenols (both pure molecules and in mixtures as plant polyphenolic extracts) have been suggested as supportive therapies for gut health. Here, we provide the first evidence that PPE could have direct beneficial effects on barrier function in human intestinal epithelial Caco-2 cells and in rat colons. Furthermore, we found that the regulation of the TJ assembly is related to the activation of AMPK and ERK signaling.

It is widely known that intact intestinal barrier function is extremely important for the maintenance of a healthy gut and pathogen prevention. Human intestinal epithelial Caco-2 cells are widely used as an *in vitro* model for evaluating intestinal barrier function. We found that PPE could significantly increase TJ integrity by increasing TER and decreasing the LY permeability of cultured Caco-2 monolayers (Figure 1A). The intestinal barrier function can be enhanced or disturbed by a wide range of substances [9,31,32], including dietary nutrients, such as amino acids, polysaccharides, and polyphenols, or derivatives, such as SCFA [8]. Polyphenol-rich foods such as green tea, cocoa, and fruits can improve gut barrier function [33,34,35], suggesting that propolis, which is also rich in polyphenols, may also promote intestinal barrier integrity. Polyphenolic constituents such as quercetin and kaempferol, which have been demonstrated to enhance barrier function [21,36], are also present in our PPE samples [19], and are likely to contribute to the beneficial effects on TER observed in our study. As TJ protein expression and distribution patterns are the major determinants of intestinal barrier function, it is likely that the abundant polyphenols in PPE have acted to improve barrier function by influencing the TJ proteins. Therefore, additional studies are necessary to clarify the roles of these biological constituents in PPE.

TJ formation and assembly involve a complex of proteins that are regulated by several signaling cascades, including kinases, phosphatases, or G proteins [6]. A significant body of evidence has recently accumulated indicating that crosstalk between diverse signaling pathways regulates the formation, as well as the disassembly, of TJs. AMPK regulates several important membrane-transport proteins [37]. It is a metabolic-sensing kinase involved in ATP generation in cells. According to recent studies, activated AMPK promotes TJ formation. The phosphatidylinositol 3-kinase/Akt pathway is known as an important regulator of intestinal cell proliferation and intestinal permeability [38,39]. Nevertheless, Li *et al*. found that inhibition of the PI3K/Akt pathway did not alter the TER, permeability, or expression levels of claudin-1 in Gln-supplemented cells, which is consistent with our findings [40].

The intracellular signal transduction MAPK family is one of the best-characterized intracellular signaling pathways [41]. ERK, the c-Jun *N*-terminal kinase (JNK), and the p38 subfamilies are the most extensively characterized MAPK members. Using human Caco-2 cells, we found that PPE treatment led to the phosphorylation of ERK1/2 and p38, but not JNK. This was consistent with our previous study using murine RAW 264.7 macrophages, but inconsistent with studies of neuron-like PC12 and fibroblast cells [42,43]. It should be noted that the MAPK pathway has a complicated role in TJ regulation and conflicting results have been reported. Kinugasa *et al*. found that ERK1/2 activation leads to increased TER intestinal epithelial cells and the upregulation of TJ claudin-1 and 2 mRNA expressions [44]. Similar results have been found using renal epithelial LLC-PK1 cells [45]. Nevertheless, tumor necrosis factor (TNF-α), the major proinflammatory cytokine in IBD, increases intestinal permeability and will result in the rapid activation of ERK1/2 and a myosin light chain kinase-dependent opening of intestinal TJ [46]. Wu *et al*. found that the downregulation of tight junction proteins might be related to p38 MAPK activation in a clopidogrel-induced gastric injury model [47]. Here, we found that the PPE-induced changes in TER values by PPE were prevented in Caco2 cells where AMPK and ERK1/2 signaling was inhibited. Nevertheless, the effects of these inhibitors on the tight junction proteins still need to be clarified in the future, considering that pharmacological inhibitors such as those used in the present study may inhibit other kinases. Interestingly, the inhibition of p38-MAPK showed contradictory effects, since TER values showed greater increases. These results strongly suggest that p38 MAPK activation has a negative role in the regulation of Caco-2 cell permeability. To our knowledge, these data provide direct evidence that PPE activates ERK1/2 and AMPK signaling in intestinal epithelial cells.

It should be noted that PPE-mediated modulation of barrier function was not related to cytotoxicity (as demonstrated by the cell counting kit-8 cell viability assay, Figure 1C). Some studies have suggested that increases in TER might be generated by a decreased cell number, as both cell density and intracellular spaces in the monolayer increase [31]. In our study, immunofluorescence staining of cells allowed us to show that the spatial expression of the occludin and ZO-1 proteins had a similar junction pattern in treated cells and to also show that cell density was also unaffected by treatment. Moreover, using transmission electron microscopy, we first showed that PPE-treated cells had normal tissue structures, unlike the tBHP damage control cells. We inferred that increases in TER as well as other barrier function enhancements by PPE were likely not due to alterations in cell number or size.

In our previous *in vitro* studies using murine macrophages (Raw264.7) we found that PPE concentration up to 10 μg/mL was effective but concentrations above this were toxic to the cells [18,19]. In this study, the highest concentration of propolis applied to the human intestinal epithelial cells was 50 μg/mL as this dose was the most effective for increasing intestinal barrier function. Since the samples we used are the same in these two studies, we inferred that the intestinal epithelial cells have higher tolerance to PPE than the macrophages. In our previous animal studies, propolis was administered by gavage at a dose of 100 mg/kg body weight to the rats/mice. This dosage was safe and effective against endotoxin induced mouse acute lung injury and alleviated hepatorenal lesion in diabetic rats [19,24]. The incorporation rate of propolis into feed (0.3% *w/w*) was designed for the rats to achieve an approximate 100 mg/kg body weight dose once the estimated feed intake levels were taken into account. This dosage selection rationale also provides us a reference for the future potential clinical trials of propolis.

## 5. Conclusions

Overall, PPE enhanced the barrier function in the Caco-2 cell monolayer and promoted colonic occludin mRNA expression in rats fed diets containing PPE. Additionally, the effects of PPE on the regulation of the intestinal barrier function were mediated, at least in part, by AMPK and ERK1/2 activation, and were negatively regulated by p38 MAPK signaling. These results provide new insights into the molecular mechanisms underlying the beneficial effects of polyphenol-rich foods (e.g., bee propolis) on gut health and have important implications for human IBD prevention/treatment.

## Supplementary Materials

The following are available online at http://www.mdpi.com/2072-6643/8/5/272/s1, Supplementary methods, Table S1: Composition of the diet, Figure S1: Effects of tBHP on treatment on the distribution of tight junction proteins (ZO-1 and occludin), as well as tight junction morphological ultrastructure in Caco-2 cells, Figure S2: Time course effect of PPE treatment on the activations of AMPK, Akt, ERK and p38, Figure S3: Effects of oral administration of PPE on rat colon morphology.
