# Supplementary material for: Polyphenol-Rich Propolis Extracts Strengthen Intestinal Barrier Function by Activating AMPK and ERK Signaling
Source: Nutrients. 2016 May 7;8(5):272. doi: 10.3390/nu8050272 (PMC4882685; doi:10.3390/nu8050272)
Supplement: Supplementary file 1 [file nutrients-08-00272-s001.docx]

Supplementary Materials: Polyphenol-Rich Propolis Extracts Strengthen Intestinal Barrier Function by Activating AMPK and ERK Signaling

Kai Wang, Xiaolu Jin, Yifan Chen, Zehe Song, Xiasen Jiang, Fuliang Hu, Michael A. Conlon, David L. Topping

Supplementary Methods

1.1. Histology Examination

Colon tissues were fixed with formalin (10%) and then embedded in paraffin. Between six and ten 4-μm-thick sections were prepared in a noncontiguous manner and stained with hematoxylin and eosin stain (HE). HE-stained slides were visualized under a light microscope (Nikon Eclipse 80i, Tokyo, Japan) and images were taken using an attached camera.

1.2. Electron Microscopy Analysis

1.2.1. Transmission Electron Microscopy

The Caco-2 cellular monolayer was fixed in glutaraldehyde (2.5%) and 1% osmium tetroxide (1%) in 0.01 M phosphate buffer (pH 7.0). Samples were then embedded in Epon 812 resin overnight and dried with gradient acetone, following the manufacturer’s instructions (SPI-EM, Division of Structure Probe, Westchester, NY, USA). After obtaining ultrathin sections (70 nm), uranium acid and lead citrate were used for drying, and samples were observed under a TEM (H-7650, Hitachi, Tokyo, Japan).

1.2.2. Scanning Electron Microscopy

For the SEM analysis, 1 cm of distal colon tissue from each rat was rinsed three times with PBS (pH 7.0). Then, the colon tissue samples were fixed in glutaraldehyde (2.5%) for 24 h at 4 °C. Specimens were washed three times for 15 min with 0.1 M phosphate buffer, pH 7.0. Samples were dehydrated using isoamyl acetate, coated with gold, and observed using a SEM (S-3000N, Hitachi Ltd., Tokyo, Japan).

**Table S1.** Composition of the diet.

| **Components** | **g/kg Diet** |
| --- | --- |
| Casein | 250 |
| Cornstarch | 350 |
| Sucrose | 100 |
| Fat Blend (Canola and Palm oils) | 200 |
| Wheat bran | 50 |
| l-Cystine | 3 |
| Choline bitartrate | 2.5 |
| Vitamins (AIN 93) | 10 |
| Minerals | 35 |
| Tert-butyl hydroquinol | 0.014 |


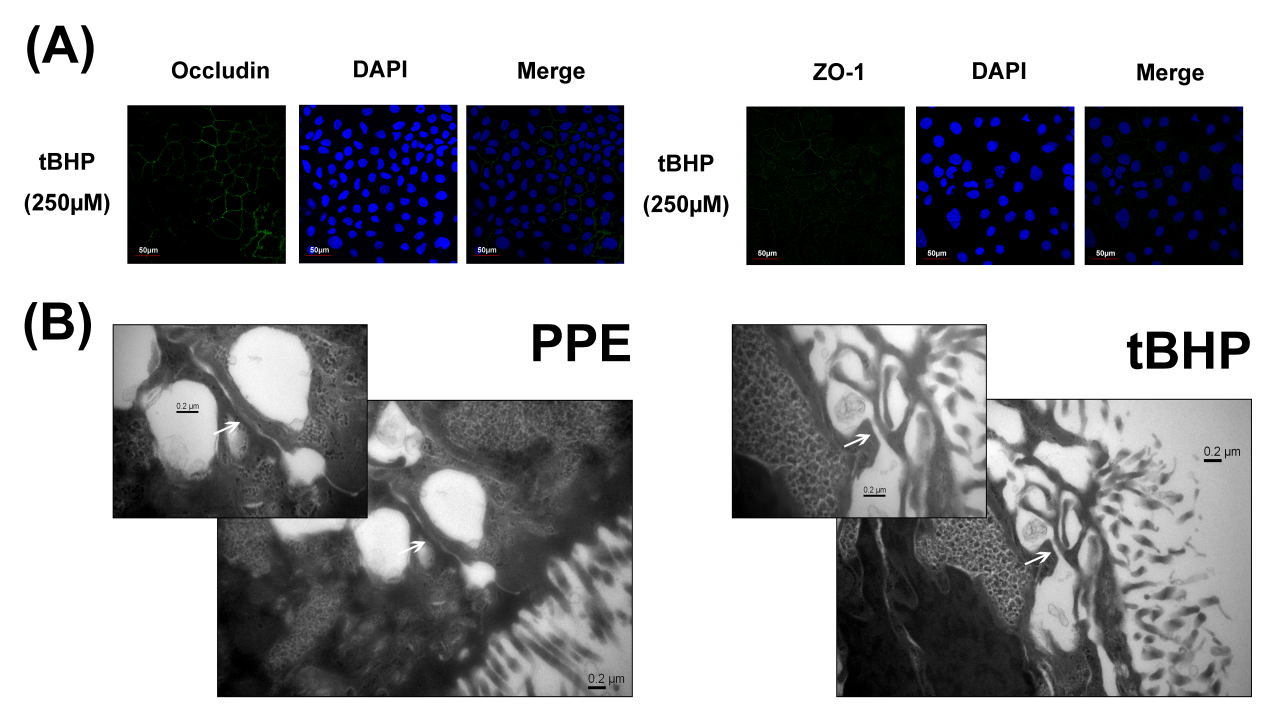


**Figure S1.** Effects of tBHP on treatment on the distribution of tight junction proteins (*ZO-1* and *occludin*), as well as the tight junction morphological ultrastructure in Caco-2 cells. (**A**) Confocal microscopy images of immuno-stained tight junction proteins in confluent Caco-2 cells untreated or treated with tBHP (250 µM) for 12 h. Representative confocal microscopy images were obtained by fluorescent microscopy from three independent experiments after immunofluorescence staining of *ZO-1* and *occludin*. 4′,6-Diamidino-2-phenylindole (DAPI) staining was performed to identify nuclei; (**B**) Transmission electron microscopy (TEM) images showed the effects of tBHP or PPE treatment on the morphological ultrastructure of tight junction in Caco-2 cell monolayers. Caco-2 cell monolayers were grown on 12-Costar Transwell filters for 14 days and exposed to either PPE (50 µg/mL, left panel) or tBHP (250 µM, right panel) for 48 h. Cells were then fixed and processed for TEM. The tight junctions between cells are indicated by white arrows.

**
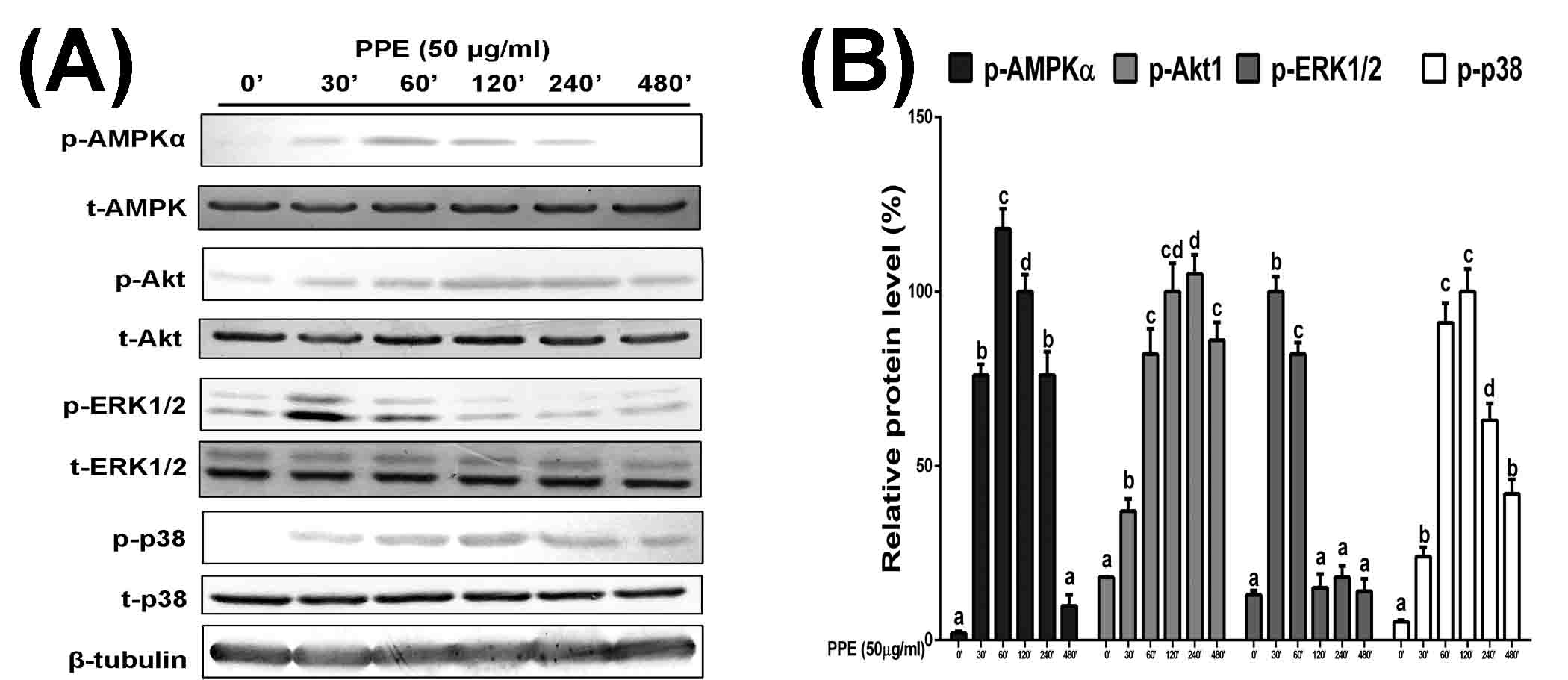
**

**Figure S2.** Time course effect of PPE treatment on the activations of AMPK, Akt, ERK, and p38. Caco-2 cells were grown to confluence on 6-well plates and treated with PPE (50 µg/mL) for the indicated periods. (**A**) Whole cell lysates were collected and further subjected to an immunoblot analysis. Specific antibodies were used to detect the expression of phospho-AMPKα, total-AMPK, phospho-Akt, total-Akt, phospho-ERK1/2, total-ERK1/2, phospho-p38, and total-p38. β-Tubulin was used as a loading control. Representative Western blots are shown from three independent experiments; (**B**) The intensity of corresponding bands was measured by densitometry and normalized to β-tubulin. The values are the means ± SD (*n* = 3). Means sharing the same letter are not significantly different from each other (*p* < 0.05).


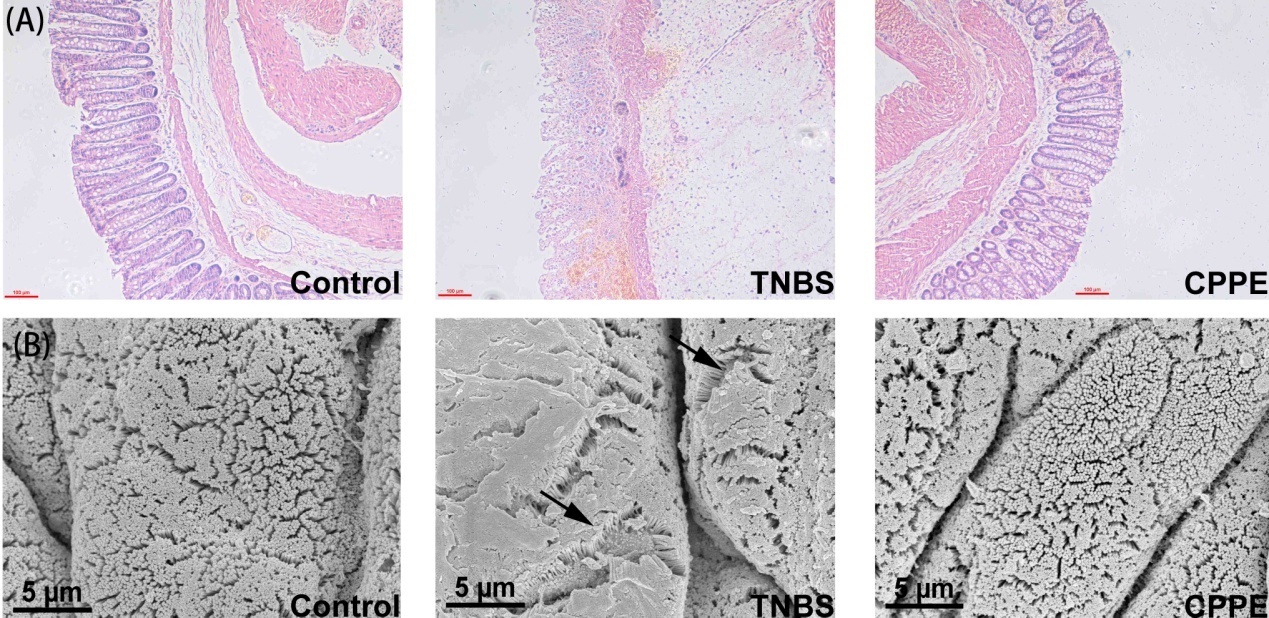


**Figure S3.** Effects of oral administration of PPE on rat colon morphology. Distal colon tissues were collected from rats that were administered the control diet or control diet containing PPE (0.3% w/w). TNBS was injected in the rats with control diets and set as the colitis control. (**A**) Light microscope images of the distal colon epithelium (bar is 100 μm); and (**B**) scanning electron micrographs of the distal colon epithelium (bar is 5 μm). Images were taken from distal colon tissues and are representative of six rats.
